# Melatonin Alleviates Liver Apoptosis in Bile Duct Ligation Young Rats

**DOI:** 10.3390/ijms17081365

**Published:** 2016-08-20

**Authors:** Jiunn-Ming Sheen, Yu-Chieh Chen, Mei-Hsin Hsu, You-Lin Tain, Ying-Hsien Huang, Mao-Meng Tiao, Shih-Wen Li, Li-Tung Huang

**Affiliations:** 1 Department of Pediatrics, Kaohsiung Chang Gung Memorial Hospital and Chang Gung University College of Medicine, 123 Ta-Pei Road, Niao Sung, Kaohsiung 833, Taiwan; ray.sheen@gmail.com (J.-M.S.); a03peggy@adm.cgmh.org.tw (M.-H.H.); tainyl@hotmail.com (Y.-L.T.); yhhuang@cgmh.org.tw (Y.-H.H.); tmm@adm.cgmh.org.tw (M.-M.T.); violet7053@gmail.com (S.-W.L.); 2 Graduate Institute of Clinical Medical Sciences, Chang Gung University College of Medicine, Linkou, Taoyuan 333, Taiwan

**Keywords:** bile duct ligation, melatonin, apoptosis, endoplasmic reticulum, mitochondria

## Abstract

Bile duct ligation (BDL)-treated rats display cholestasis and liver damages. The potential protective activity of melatonin in young BDL rats in terms of apoptosis, mitochondrial function, and endoplasmic reticulum (ER) homeostasis has not yet been evaluated. Three groups of young male Sprague-Dawley rats were used: one group received laparotomy (Sham), a second group received BDL for two weeks (BDL), and a third group received BDL and intraperitoneal melatonin (100 mg/day) for two weeks (BDL + M). BDL group rats showed liver apoptosis, increased pro-inflamamtory mediators, caspases alterations, anti-apoptotic factors changes, and dysfunction of ER homeostasis. Melatonin effectively reversed apoptosis, mainly through intrinsic pathway and reversed ER stress. In addition, in vitro study showed melatonin exerted its effect mainly through the melatonin 2 receptor (MT2) in HepG2 cells. In conclusion, BDL in young rats caused liver apoptosis. Melatonin rescued the apoptotic changes via the intrinsic pathway, and possibly through the MT2 receptor. Melatonin also reversed ER stress induced by BDL.

## 1. Introduction

Bile duct ligation (BDL) is extensively used as a model of acute and chronic liver injury accompanied by cholestasis [1,2,3,4]. Obstructive jaundice occurs immediately and evolves to cirrhosis in four to six weeks [5,6]. BDL in rats is characterized by both peripheral and central inflammation [7,8]. It is well documented that oxidative stress is implicated in the pathogenesis of liver damage and systemic organ dysfunction in young and adult rats after BDL [1,4,7,9,10].

Apoptosis, which is also named programmed cell death, is strongly associated with mitochondrial function and is considered an important component of various physiological processes including normal cell turnover, adequate development and function of the immune system, embryonic development, hormone-dependent atrophy, and chemical substance-induced cell death [11]. Melatonin has been shown to be able to attenuate hepatocyte apoptosis in BDL adult rats [12]; however, the underlying mechanisms are not clear. Mitochondrial structures are highly susceptible to oxidative injury and mitochondrial damages take an important role in cell death [13]. Melatonin is effective in protecting liver mitochondrial damages in diabetic obese rats [14].

Endoplasmic reticulum (ER) plays a pivotal role in biosynthesis and maturation of proteins, synthesis of lipids, adjustment of calcium, and preservation of cell homeostasis [15]. A variety of disorders such as hypoxia, glucose starvation, and oxidative stress may lead to endoplasmic reticulum (ER) disorder, which can provoke ER stress. Recently, increasing studies show ER stress is a salient feature of numerous liver diseases [16,17]. Recently, melatonin is shown to be effective in reducing ER stress-induced hepatic steatosis [18] and tetrachloride-induced liver fibrosis [19].

Melatonin is a strong antioxidant [20,21] and confers ability to protect mitochondrial from damage [22,23]. Melatonin is widely used in various pre-clinical studies [23] with doses ranging from 0.1 to 100 mg/kg/day for various days via different routes. Though the beneficial effects of melatonin in BDL liver damages are well delineated [1,23,24,25], the underlying molecular mechanisms are largely unexplored. In the present study, we aimed to evaluate the therapeutic effect of melatonin in alleviating liver apoptosis in BDL young rats and focused on apoptosis pathway, mitochondrial function, and ER stress.

## 2. Results

### 2.1. Bile Duct Ligation (BDL) Resulted in Increased Hepatic Apoptosis

**Figure 1:**
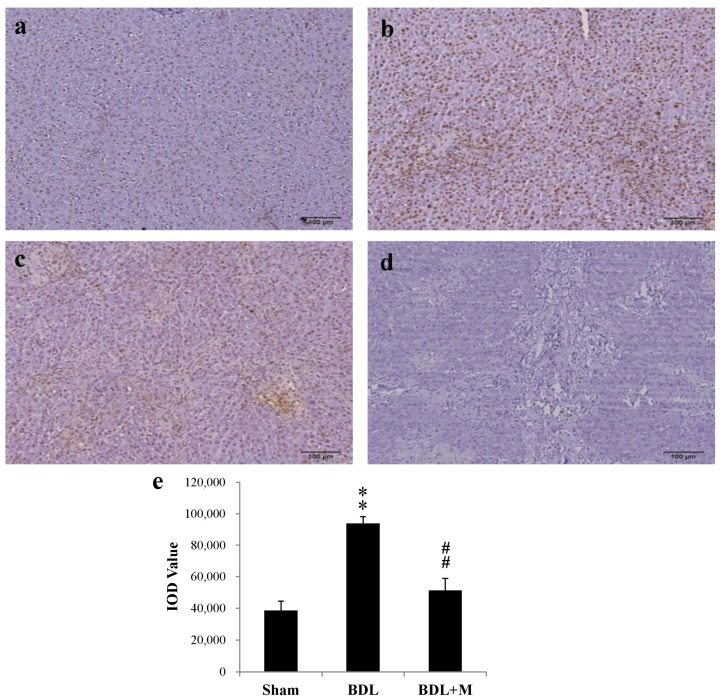
Representative Terminal deoxynucleotidyl transferase dUTP nick end labeling (TUNEL) staining of liver in bile duct ligation (BDL) rats without and with melatonin treatment. TUNEL staining was increased in BDL rat and this was significantly reduced by melatonin treatment: (**a**) Sham; (**b**) BDL; (**c**) BDL + M; (**d**) negative control; and (**e**) integrated optical density (IOD) value of TUNEL stain. Data are shown as mean ± standard error of mean (SEM). ** *p* < 0.01 vs. Sham; ## *p* < 0.01 vs. BDL.

BDL rats had higher plasma total and direct bilirubin, aspartate aminotransferase (AST) and alanine aminotransferase (ALT) levels than the Sham group (Table 1). Melatonin treatment caused reduction of direct bilirubin levels (Table 1). As shown in Figure 1, BDL resulted in increased liver apoptosis and melatonin treatment reduced the process, which was evidenced by Terminal deoxynucleotidyl transferase dUTP nick end labeling (TUNEL) stain.

### 2.2. BDL Increased the Mrna Expression of Proinflammatory Mediators and Melatonin Treatment Altered the Changes

**Figure 2:**
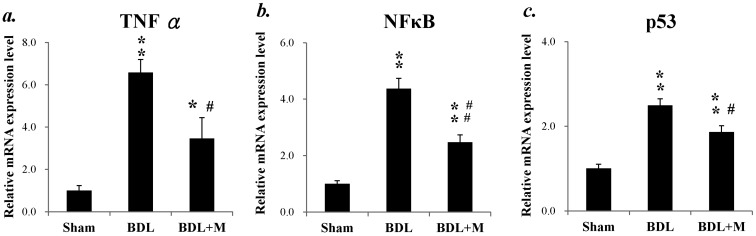
Melatonin treatment reduced the mRNA expression of pro-inflammatory mediators induced by BDL: (**a**) BDL induced increased mRNA expression of TNF-α and melatonin treatment decreased the effect; (**b**) BDL induced increased mRNA expression of nuclear factor-kappa B (NFκB) and melatonin treatment decreased the effect; and (**c**) BDL induces increased mRNA expression of p53 and melatonin treatment decreased the effect. Significant difference among three groups was analyzed by one-way ANOVA followed by Bonferroni post hoc. All data are shown as mean ± SEM. * *p* < 0.05 vs. Sham; ** *p* < 0.01 vs. Sham; # *p* < 0.05 vs. BDL; ## *p* < 0.01 vs. BDL.

As shown in Figure 2, BDL resulted in increased mRNA expression of tumor necrosis factor-α (TNF-α) mRNA (F (2, 25) = 16.982, *p* < 0.001), nuclear factor-kappa B (NFκB) mRNA (F (2, 22) = 63.263, *p* < 0.001), and p53 mRNA (F (2, 22) = 35.768, *p* < 0.001). Melatonin treatment caused significant reduction of the mRNA expression of all the three pro-inflammtory mediators.

### 2.3. BDL Induced Liver Apoptosis via the Caspase-Dependent Pathway

**Figure 3:**
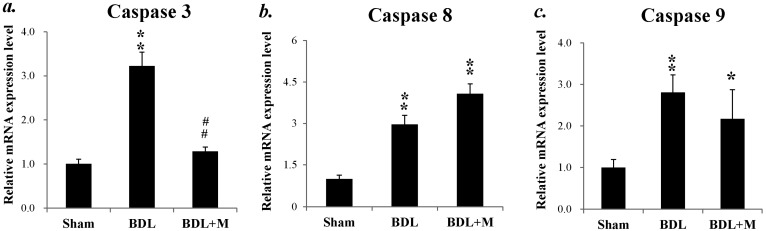
mRNA expression of caspase 3, 8 and 9: (**a**) expression of caspase 3 mRNA was increased in BDL group and down-regulated in BDL + M group; (**b**) BDL group had higher caspase 8 mRNA than Sham group; and (**c**) caspase 9 mRNA was increased in BDL and BDL + M groups. Significant difference among three groups was analyzed by one-way ANOVA followed by Bonferroni post hoc. All data are shown as mean ± SEM. * *p* < 0.05 vs. Sham; ** *p* < 0.01 vs. Sham; ## *p* < 0.01 vs. BDL.

Caspases can be activated through intrinsic pathway or extrinsic pathway and lead to apoptosis. We analyzed the caspase mRNA expressions because previous reports have shown that the caspase mRNA levels may be increased in the apoptotic process [26,27,28]. As shown in Figure 3, BDL increased caspase 3, 8, and 9 mRNA expressions (caspase 3 mRNA (F (2, 22) = 47.488, *p* < 0.001); caspase 8 mRNA (F (2, 24) = 48.954, *p* < 0.001); caspase 9 mRNA (F (2, 24) = 5.683, *p* < 0.01)). Increased caspase 3, 8, and 9 mRNA expressions indicated that BDL in young rats led to liver apoptosis. Bonferroni post hoc showed melatonin treatment effectively down-regulated the mRNA expression of caspase 3, (BDL vs. BDL + M, *p* < 0.01).

**Figure 4:**
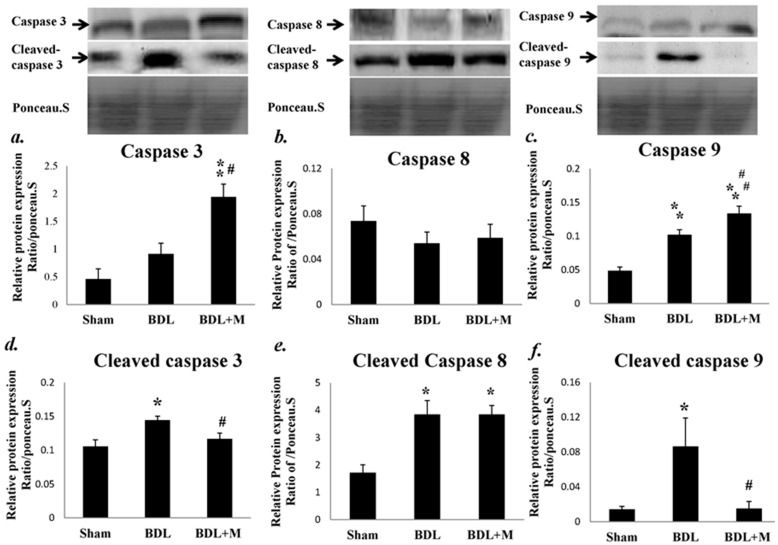
Caspase 3 (**a**) and caspase 8 (**b**) protein expressions were not affected by BDL. BDL for two weeks increased: caspase 9 (**c**); cleaved caspase 3 (**d**); cleaved caspase 8 (**e**); and cleaved caspase 9 protein expression (**f**). Melatonin treatment decreased cleaved caspase 3 and cleaved caspase 9. Significant difference among three groups was analyzed by one-way ANOVA followed by Bonferroni post hoc. All data are shown as mean ± SEM. * *p* < 0.05 vs. Sham; ** *p* < 0.01 vs. Sham; # *p* < 0.05 vs. BDL; ## *p* < 0.01 vs. BDL.

In addition, Western blot revealed increased protein expression of cleaved caspase 3, 8 and 9 in BDL group (cleaved caspase 3 (F (2, 14) = 6.559, *p* = 0.010); cleaved caspase 8 (F (2, 14) = 15.469, *p* < 0.001); cleaved-caspase 9 (F (2, 13) = 6.431, *p* = 0.011)), and melatonin treatment significantly reduced the protein expression of cleaved caspase 3 and cleaved caspase 9 (BDL vs. BDL + M, both *p* < 0.05), but had no significant effects on the protein expression of cleaved caspase 8 (Figure 4). The discrepancy of mRNA and protein levels of caspase 3 in BDL + M group could be due to downregulation of miRNA targeting mRNA or a feedback loop inhibits further transcription process.

**Figure 5:**
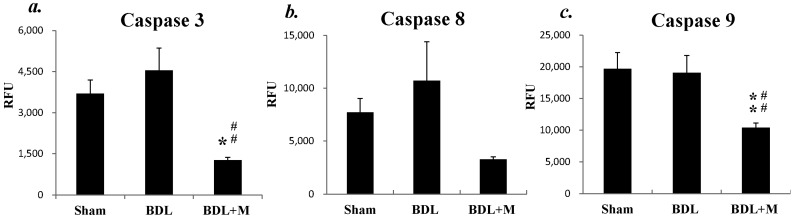
BDL for two weeks did not affect caspase 3 (**a**), caspase 8 (**b**), or caspase 9 (**c**) activities. Melatonin treatment decreased caspase 3 and caspase 9 activities. Significant difference among three groups was analyzed by one-way ANOVA followed by Bonferroni post hoc. All data are shown as mean ± SEM. * *p* < 0.05 vs. Sham; ** *p* < 0.01 vs. Sham; ## *p* < 0.01 vs. BDL. RFU: Relative fluorescence units.

Moreover, fluorescent activity study for caspase activity found that melatonin treatment effectively reduced caspase 3 and caspase 9 activities (caspase 3 activity (F (2, 15) = 9.440, *p* = 0.002); caspase 9 activity (F (2, 34) = 7.413, *p* = 0.002)), but had no significant effects on the caspase 8 activity (Figure 5). The above findings indicated that BDL altered the apoptotic pathway through a caspase-dependent manner and melatonin might exert its therapeutic function through a caspase-dependent, chiefly the intrinsic, pathway.

### 2.4. Melatonin Blocked the Apoptotic Signal Mediation Mainly through the Intrinsic Pathway Induced by BDL

**Figure 6:**
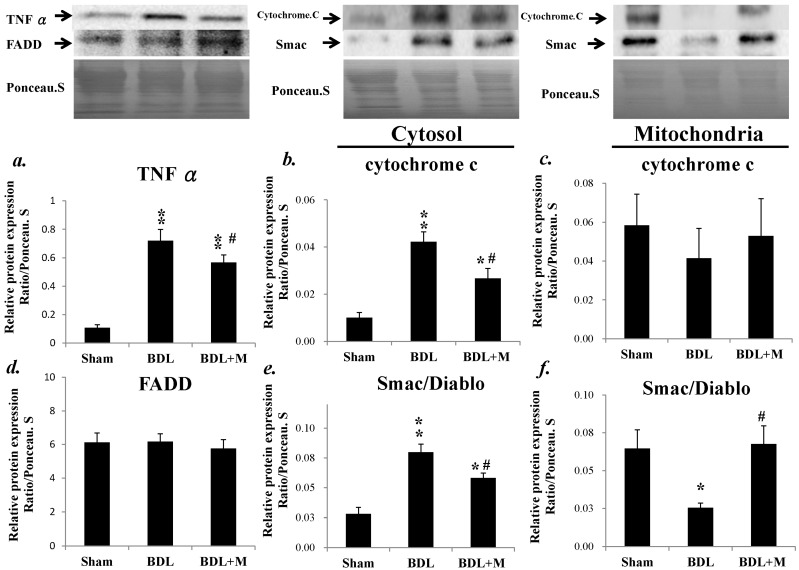
(**a**) BDL for two weeks increased TNF-α protein expression while melatonin treatment reduced this effect; (**b**) cytosolic cytochrome c was highly expressed in response to BDL and melatonin treatment rescued this process; (**c**) mitochondrial cytochrome c was decreased in response to BDL; (**d**) Fas-Associated protein with Death Domain (FADD) was not affected by BDL or melatonin treatment; (**e**) cytosolic second mitochondria-derived activator of caspase/direct inhibitor of apoptosis-binding protein with low pl (Smac/Diablo) was increased after BDL and melatonin effectively reversed the effect; and (**f**) mitochondrial Smac/Diabo change was not reversed by melatonin treatment. Significant difference among three groups was analyzed by one-way ANOVA followed by Bonferroni post hoc. All data are shown as mean ± SEM. * *p* < 0.05 vs. Sham; ** *p* < 0.01 vs. BDL; # *p* < 0.05 vs. BDL.

Western blot revealed increased TNF-α after BDL and melatonin treatment significantly reversed the change (F (2, 23) = 28.673, *p* < 0.001; BDL vs. Sham, *p* < 0.01; BDL vs. BDL + M, *p* < 0.05). Further examination of the extent of the involvement of the extrinsic pathway in the apoptotic process after BDL, Fas-Associated protein with Death Domain (FADD) was examined. However, FADD was not affected by BDL or melatonin treatment (F (2, 23) = 0.230, *p* = 0.796). To explore the intrinsic pathway, we examined cytochrome c and Second mitochondria-derived activator of caspase/direct inhibitor of apoptosis-binding protein with low pl (Smac/Diablo) and found cytosolic cytochrome c and Smac/Diablo were increased in response to BDL and melatonin treatment successfully rescued the process (cytochrome c, (F (2, 15) = 6.257, *p* = 0.011; BDL vs. BDL + M, *p* < 0.05); Smac/Diablo (F (2, 15) = 16.841), *p* < 0.001; BDL vs. BDL + M, *p* < 0.05) (Figure 6).

**Figure 7:**
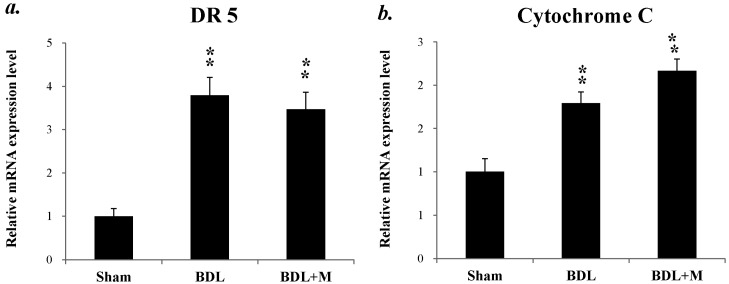
(**a**) BDL and melatonin treatment induced the up-regulation of TNF-Related Apoptosis-Inducing Ligand Receptor 2/death receptor 5 (TRAIL-R2/DR 5) and melatonin treatment showed no effect on it; (**b**) BDL increased cytochrome c mRNA expression and melatonin treatment showed no effect on it. Significant difference among three groups was analyzed by one-way ANOVA followed by Bonferroni post hoc. All data are shown as mean ± SEM. ** *p* < 0.01 vs. BDL.

Besides, we studied apoptosis-related genes, TNF-Related Apoptosis-Inducing Ligand Receptor 2 (TRAIL-R2)/death receptor 5 (DR5) and cytochrome c, which represented extrinsic and intrinsic pathway, respectively. As shown in Figure 7, BDL induced up-regulation of DR5 and cytochrome c mRNA expression (DR5 (F (2, 24) = 34.866, *p* < 0.001); cytochrome c (F (2, 24) = 18.621, *p* < 0.001)). However, melatonin treatment had not effect in lowering these alterations. Taken together, melatonin rescued the liver apoptotic process mainly through the intrinsic pathway.

### 2.5. Anti-Apoptotosis Factors Change in Response to BDL and Melatonin Treatment

**Figure 8:**
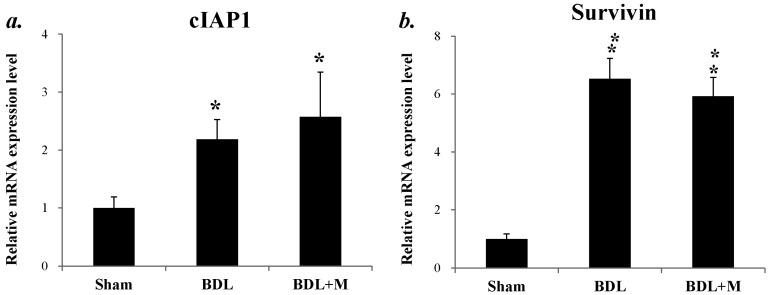
mRNA expression of anti-apoptosis factors. Cellular inhibitor of apoptosis (cIAP1) (**a**) and survivin mRNA (**b**) were increased in BDL rats. Significant difference among three groups was analyzed by one-way ANOVA followed by Bonferroni post hoc. All data are shown as mean ± SEM. * *p* < 0.05 vs. Sham; ** *p* < 0.01 vs. Sham.

Cellular inhibitor of apoptosis (cIAP1) and survivin mRNA were increased in response to BDL, but melatonin had no significant effects (cIAP1 (F (2, 24) = 5.574, *p* < 0.010); surviving (F (2, 24) = 68.549, *p* < 0.001); BND vs. BDL + M, all *p* > 0.05) (Figure 8).

**Figure 9:**
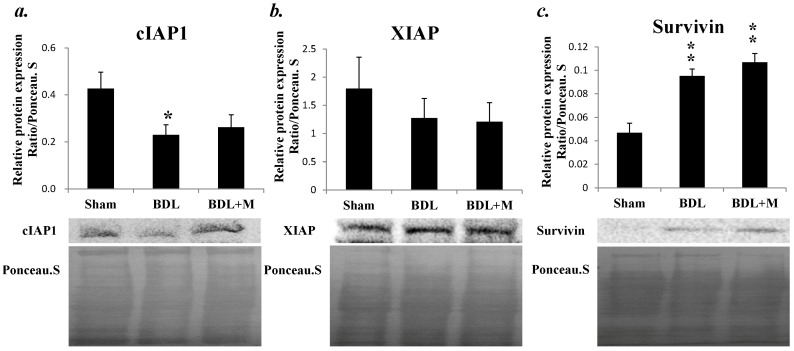
(**a**) Western blot revealed decreased level of cIAP1 in BDL rats; (**b**) both BDL and melatonin treatment did not alter the X-linked inhibitor of apoptosis protein (XIAP) expression; and (**c**) survivin was increased in BDL and BDL + M groups. Significant difference among three groups was analyzed by one-way ANOVA followed by Bonferroni post hoc. All data are shown as mean ± SEM. * *p* < 0.05 vs. Sham; ** *p* < 0.01 vs. Sham.

As for apoptosis related inhibitors, such as cIAP1 and X-linked inhibitor of apoptosis protein (XIAP), BDL only decreased the protein expression of cIAP1 (F (2, 23) = 4.004, *p* = 0.032) and melatonin treatment revealed no significant change in cIAP1. On the other hand, survivin protein expression was increased in BDL and BDL + M groups (F (2, 23) = 20.937, *p* < 0.001) (Figure 9). Together, these results suggested BDL and melatonin treatment might also alter the anti-apoptotic process.

### 2.6. Endoplasmic Reticulum (ER) Stress in BDL Rat

**Figure 10:**
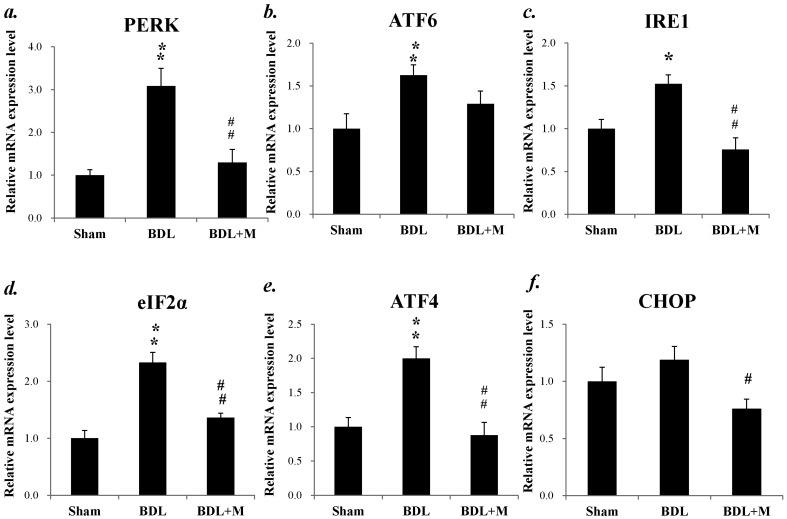
(**a**) Increased mRNA expression of protein kinase RNA-like endoplasmic reticulum kinase (PERK) in BDL rats and responded well to melatonin treatment; (**b**) increased activating transcription factor 6 (ATF6) mRNA expression in BDL rats; (**c**) increased inositol-requiring enzyme 1 (IRE1) mRNA expression and responded to melatonin treatment; (**d**) increased eukaryotic translation initiation factor 2α (eIF2α) mRNA expression and responded to melatonin treatment; (**e**) increased activating transcription factor 4 (ATF4) mRNA expression and responded to melatonin treatment; and (**f**) BDL + M group had lower CCAAT-enhancer-binding protein homologous protein (CHOP) mRNA expression than BDL group rats. Significant difference among three groups was analyzed by one-way ANOVA followed by Bonferroni post hoc. All data are shown as mean ± SEM. * *p* < 0.05 vs. Sham; ** *p* < 0.01 vs. Sham, # *p* < 0.05 vs. BDL; ## *p* < 0.01 vs. BDL.

mRNA expression of the three key regulators of ER homeostasis, protein kinase RNA-like ER kinase (PERK), inositol-requiring enzyme 1 (IRE 1) and activating transcription factor 6 (ATF6) were all increased in BDL group (PERK mRNA (F (2, 23) = 13.431, *p* < 0.001); IRE1 mRNA (F (2, 21) = 8.742, *p* = 0.002); ATF6 mRNA (F (2, 23) = 4.246, *p* = 0.027)). Bonferroni post hoc analysis revealed that PERK and IRE 1 mRNA expression were decreased after melatonin treatment (BDL vs. BDL + M, all *p* < 0.01). Moreover, further tests for the downstream regulators of ER homeostasis including CCAAT-enhancer-binding protein homologous protein (CHOP), eukaryotic translation initiation factor 2α (eIF2α) and activating transcription factor 4 (ATF4) revealed significant increases of eIF2α and AT4 mRNA expression in BDL rats (eIF2α mRNA (F (2, 23) = 23.894, *p* < 0.001); ATF4 mRNA (F (2, 23) = 9.641, *p* = 0.001)). Bonferroni post hoc analysis revealed that eIF2α and AT4 mRNA expression were decreased after melatonin treatment (BDL vs. BDL + M, all *p* < 0.01) (Figure 10). Taken together, BDL induced disturbance of ER homeostasis and melatonin treatment might effectively reverse the effect.

**Figure 11:**
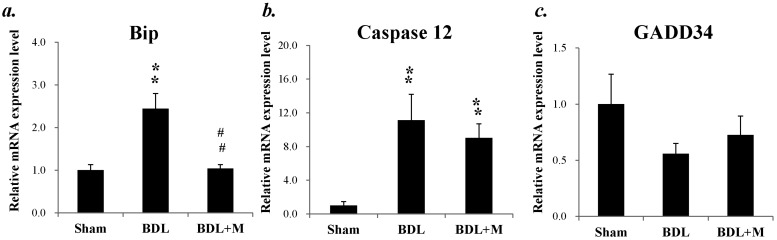
(**a**) mRNA expression of binding immunoglobulin protein (Bip) was increased in response to BDL, and melatonin treatment effectively decreased its expression; (**b**) There was increased caspase 12 mRNA expression in young BDL rats; (**c**) There was no significant difference of growth arrest and DNA-damage-inducible protein (GADD34) mRNA expression among the three groups. Significant difference among three groups was analyzed by one-way ANOVA followed by Bonferroni post hoc. All data are shown as mean ± SEM. ** *p* < 0.01 vs. Sham; ## *p* < 0.01 vs. BDL.

A shown in Figure 11, mRNA expression of Binding immunoglobulin protein (Bip) was increased in response to BDL and melatonin treatment effectively decreased its expression ((F (2, 23) = 15.660, *p* < 0.001); BDL vs. BDL + M, *p* < 0.01). To elucidate the crosstalk between apoptosis and ER homeostasis, we examined caspase 12 mRNA expression and found increased mRNA expression in BDL group, which responded well to melatonin treatment (caspase 12 mRNA (F (2, 23) = 24.087, *p* < 0.001); BDL vs. BDL + M, *p* < 0.05). Besides, As growth arrest and DNA damage-inducible protein (GADD) 34 plays a role in autoregulation (feedback mechanism) of ER stress response, we tested GADD34 mRNA expression, but found no significant difference of GADD34 mRNA among the three groups (F (2, 23) = 2.079, *p* = 0.148).

### 2.7. Melatonin Reduced Cleaved Caspase 3 Expression through the Melatonin 2 (MT2) Receptor

**Figure 12:**
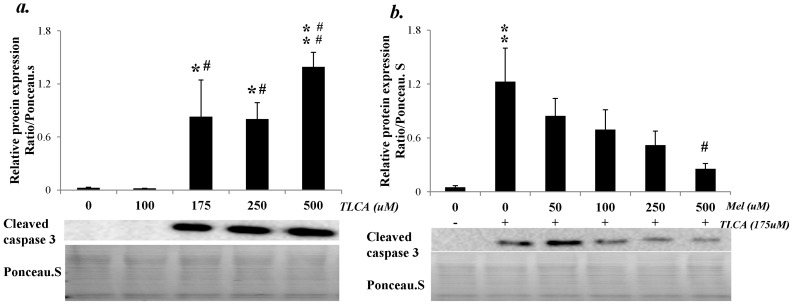

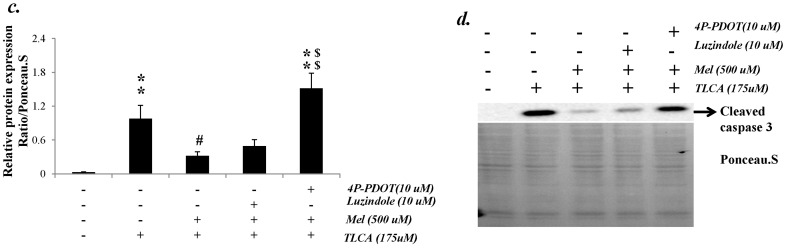
HepG2 cell culture: (**a**) There was a taurolithocholic acid (TLCA) dose-dependent protein expression of cleaved caspase 3 at six hours of incubation. * *p* < 0.05 vs. baseline; ** *p* < 0.01 vs. baseline; # *p* < 0.05 vs. 100 μM TLCA treated cells; ## *p* < 0.01 vs. 100 μM TLCA treated cells; (**b**) melatonin (Mel) effectively decreased the cleaved caspase 3 expression in a dose-dependent fashion. ** *p* < 0.01 vs. baseline ; # *p* < 0.05 vs. 175 μM TLCA with 0 μM melatonin treated cell; (**c,d**) incubating the cells with luzindole and 4-phenyl-2-propionamidotetralin (4P-PDOT) showed that melatonin reduced cleaved caspase 3 in a dose-dependent fashion, indicating melatonin acted in a caspase-dependent manner and worked through the MT2 receptor. ** *p* < 0.01 vs. control; # *p* < 0.05 vs. 175 μM TLCA only; $$ *p* < 0.01 vs. 500 μM melatonin with 175 μM TLCA treated cells.

There are three subtypes of melatonin receptors identified: MT1, MT2, and MT3 [29,30]. In mammals, the effects of melatonin are mediated mainly through MT1 and MT2 receptors. MT1 receptor is inhibited primarily by luzindole and MT2 receptor is inhibited primarily by 4-phenyl-2-propionamidotetralin (4P-PDOT). Therefore, we focused on MT1 and MT2 receptors for delineating the pathway that mediates the work of melatonin on liver apoptosis. As shown in Figure 12, there was a taurolithocholic acid (TLCA) dose-dependent expression of cleaved caspase 3 at six hours of incubation (F (4, 31) = 11.031, *p* < 0.001) (Figure 12a). Besides, melatonin effectively decreased the cleaved caspase 3 expression in a dose-dependent fashion (F (5, 34) = 4.206, *p* = 0.003) (Figure 12b). Furthermore, incubation the cells with 4P-PDOT showed melatonin reduced cleaved caspase 3 expression through MT2 receptor (F (4, 30) = 14.167, *p* < 0.001) (Figure 12c). The above findings indicated that melatonin acted in a caspase-dependent apoptosis and worked through the MT2 receptor.

## 3. Discussion

The main findings of the present study were summarized as the following: (1) BDL in young rat caused liver apoptosis; (2) melatonin rescued the apoptotic changes mainly through the intrinsic pathway and acted through the MT-2 receptor; and (3) BDL itself served as a source of ER stress and melatonin might reverse the ER stress.

In rats, two weeks of BDL represents the liver fibrogenesis stage [5]. Recently, increasing evidence support the protective role of melatonin against oxidative injury in cholestasis [31,32] and melatonin works via eliminating the oxidants [24]. In line with our previous report, melatonin partially restored liver function in BDL rats [4,25]. The present study found increased liver mRNA expression of TNF-α, NFκB and p53 in BDL young rats and melatonin treatment caused significant reduction of the mRNA expression of all the three pro-inflammatory mediators.

For liver apoptosis regulation, there is growing evidence that melatonin may directly work in the pathways of apoptosis [33,34]. The caspase-dependent apoptosis process may also be initiated via Fas ligand or oxidative stress. Molecules involving in the extrinsic (death factor-related) pathway include FADD/Tumor necrosis factor receptor type 1-associated DEATH domain protein (TRADD) and caspase 8 and intrinsic (mitochondria-related) pathway include Apaf-1 and IAPs family (caspase inhibitor family) are key regulators of apoptosis. In the present study, we tested caspase 3, 8, and 9 mRNA and protein expression in young BDL rats and found BDL altered the apoptotic pathway and melatonin had a therapeutic role.

Melatonin has been shown to be effective in protecting liver mitochondrial damages in rodent studies [14,21]. To clarify whether BDL-induced apoptosis alterations acted through intrinsic or extrinsic pathway, FADD, cytochrome c, Smac/Diablo and TRAIL-R2/DR5 were examined. We found BDL + M group rats had lower cytochrome c and Smac/Diablo protein levels as compared with BDL rats. Collectively, melatonin treatment rescues the hepatic apoptotic process in BDL young rats mainly through the intrinsic pathway. Furthermore, to understand the interaction between apoptosis and anti-apoptosis pathways induced by BDL, we tested the anti-apoptotic proteins, including cIAP1 and XIAP and found decreased level of cIAP1 in BDL rats. Both BDL and melatonin treatment did not alter the XIAP expression.

Liver apoptosis is a pathogenic event in some liver diseases, and may be related to unresolved ER stress [35]. ER homeostasis is essential for maintenance of the normal liver function. Various liver diseases are known to be lined to unresolved ER stress, which may lead to hepatocyte apoptosis. Here, we examined the essential regulators of ER homeostasis and try to evaluate the ER homeostasis and apoptosis change in BDL young rats and found increased key regulators, including Bip, PERK, IRE 1 and ATF6 mRNA expression in BDL young rats and melatonin treatment effectively reversed the effect, which indicated that BDL disrupt ER homeostasis and melatonin treatment might effectively reverse the effect. In addition, further tests for the downstream regulators of ER homeostasis found that BDL altered ER homeostasis through the CHOP-dependent pathway. Furthermore, increased caspase 12 mRNA expression in BDL rats indicated that BDL disrupted the homeostasis between mitochondria and ER.

It has been known that melatonin exerts its effect through melatonin (MT) receptor [36]. Three subtypes of melatonin receptors were identified in animals, MT1, MT2, and MT3 receptors [29], while only MT1 and MT2 but not MT3 receptors were found [29,30] in mammals. In the present study, we used melatonin receptor antagonists, luzindole (MT1 antagonist) and 4P-PDOT (MT2 antagonist), to evaluate whether melatonin acted through MT1 or MT2 receptor and found that the increased cleaved caspase 3 expression was in a taurolithocholic acid (TLCA) dose-dependent manner. Besides, melatonin effectively decreased the cleaved caspase 3 expression in a dose-dependent fashion. Furthermore, melatonin reduced cleaved caspase 3 expression in HepG2 cells incubated with 4P-PDOT, suggesting that melatonin acted through MT2 receptor.

Many trials about melatonin on various human diseases are ongoing [37,38]. Few studies used melatonin to treat human liver disease [39,40]. Celinski et al. reported that melatonin can attenuate the levels of pro-inflammatory cytokines and decrease the plasma levels of gammaglutamyl transferase, triglycerides, and low-density lipoprotein in patients who had non-alcoholic fatty liver disease [40]. Our study provides the theoretical foundation to test the therapeutic role of melatonin in children with cholestatic liver disease.

## 4. Materials and Methods

### 4.1. Subjects

This study was done under the Guidelines for Animal Experiments of Chang Gung Memorial Hospital and Chang Gung University, Taoyuan, Taiwan. We designated the day of delivery as Day 0. Male Sprague-Dawley rats postnatal day (PND) 17 ± 1 weighing about 50 g were used. We made attempts to minimize the numbers of animals used. All animals were housed in the animal care center maintained at 24 °C with a 12-h light/dark cycle. All animals had free access to water and standard chow. 

### 4.2. Experimental Procedures

All surgical procedures were done with clean surgical techniques under anesthesia with ketamine (50 mg/kg) and xylazine (23 mg/kg), as previously described [1]. In brief, the common bile duct was ligated through a midline incision then divided with double ligatures on proximal duct to induce obstructive jaundice. We designated rats that underwent sham BDL for two weeks at PND 17 ± 1 as the sham-control group (Sham) (*n* = 10). Rats that received BDL for two weeks at PND 17 ± 1 were defined as the BDL group (*n* = 10). BDL rats had daily melatonin 100 mg/kg/day intraperitoneally injected as a dose proposed by Tahan et al. [24] were designated as BDL + M (*n* = 10). All rats were sacrificed 14 days after surgery. To reduce the number of animals used, we did not perform sham group receiving melatonin because we previously found that there were no significant effects of melatonin in plasma direct and total bilirubin, AST, and ALT levels in young sham operation rats [41].

### 4.3. Plasma Biochemistry Parameters Measurement

We collected blood samples by cardiocentesis. Plasma was analyzed for aspartate aminotransferase (AST), alanine aminotransferase (ALT), and total and direct bilirubin as we previously reported [1]. 

### 4.4. Liver Histopathology

TUNEL stain was done as we previously reported [42]. TUNEL stain among the three groups was compared under 100× magnification to calculate the integrated optical density (IOD) using Image Pro Plus, Version 6.0 (Media Cybernetics, Inc., Rockville, MD, USA) [43].

### 4.5. Western Blot

We performed Western blot analysis on liver tissue as previously described [25]. The parameters examined include FADD, TRADD, Fas, and IAP family including cIAP1, XIAP, and cytochrome c. We verified the purity of the mitochondrial fraction by the selective expression of cytochrome c oxidase subunit IV, which is a mitochondrial inner membrane specific protein.

### 4.6. Quantitative Real-Time Polymerase Chain Reaction (PCR) analysis

PCR analysis was performed as we reported previously [25]. Table 2 shows the primer sequences investigated in this study. We ran the whole sample in duplicate (2.5 µL of cDNA per well in a 96-well format). We used the comparative threshold cycle (*C*_t_) method to quantify the relative gene expression. The averaged *C*_t_ was subtracted from the corresponding averaged 18S value for each sample to result in Δ*C*_t_. We obtained ΔΔ*C*_t_ by subtracting the average control ∆*C*_t_ value from the average experimental Δ*C*_t_. We established the fold increase by calculating 2^−ΔΔ*C*t^ for experimental vs. control samples.

### 4.7. HepG2 Cell Culture

HepG2 cells (human hepatoma, ATCC HB-8065) were grown at 37 °C under 5% of CO_2_ incubator. The medium used was minimal essential medium (pH 7.4) containing 10% fetal bovine serum, 1% nonessential amino acids, 2 mmol/L of l-glutamine, 1 mmol/L of sodium pyruvate, 100 units/mL of penicillin and 100 μg/mL of streptomycin. To explore whether melatonin exerted its effect on the caspase-dependent apoptosis pathway through MT1 or MT2, HepG2 cells were incubated with different concentrations of chenodeoxycholic acid and melatonin [44]. Furthermore, melatonin receptor antagonists, luzindole (MT1 antagonist) and 4P-PDOT (MT2 antagonist) were used to determine whether melatonin exerted its effects through melatonin receptors. 

### 4.8. Statistical Analysis

We analyzed the blood biochemistry, PCR, Western blotting, and ELISA results among the groups by one-way analysis of variance (ANOVA) with Bonferroni post hoc tests. We used mean ± standard error of mean (SEM) to express the values. Significance was defined as *p* < 0.05 for all tests.

## 5. Conclusions

The present study delineates a comprehensive view of liver apoptosis in BDL young rats and highlights the therapeutic role of melatonin in this process. BDL induces overwhelming dysregulation of liver apoptosis pathways, involving both mitochondria dysfunction and ER stress. Besides, the effect of BDL on apoptosis after BDL is mediated mainly through intrinsic pathway, which can be rescued by melatonin treatment.

## Figures and Tables

**Table 1:** Plasma liver function profiles in different experimental groups.

Groups	Sham (*n* = 10)	BDL (*n* = 10)	BDL + M (*n* = 10)
AST (IU/L)	97.8 ± 3.6	411.5 ± 21.8 **	493.6 ± 40.2 **
ALT (IU/L)	34.7 ± 1.8	108.2 ± 6.7 **	99.8 ± 7.8 **
Direct Bilirubin (mg/dL)	0.17 ± 0.02	4.71 ± 0.32 **	3.63 ± 0.39 **^,#^
Total bilirubin (mg/dL)	0.41 ± 0.28	5.63 ± 0.38 **	5.07 ± 0.50 **

Values are shown as means ± standard error of mean (SEM); AST, aspartate aminotransferase; ALT, alanine aminotransferase; BDL, bile duct ligation. ** *p* < 0.01 vs. sham ; ^#^ *p* < 0.05 vs. BDL.

**Table 2:** Primer sequences used in the Real-Time PCR.

Gene	Forward 5′–3′	Reverse 5′–3′
*Caspase-3*	GGCCGACTTCCTGTATGC	GCGCAAAGTGACTGGATG
*Caspase-8*	ACGATATTGCTGAACGTCTGG	CCGACTGATATGGAAAAGCAG
*Caspase-9*	GGAAGATCGAGAGACATGCAG	CCGTGACCATTTTCTTAGCAG
*18S*	GCGATGCGGCGGCGTTAT	AGACTTTGGTTTCCCGGAAGC
*Cytochrome c*	AACCTCCATGGTCTGTTTGG	GTCTGCCCTTTCTCCCTTCT
*cIAP1*	AGCTTGCAAGTGCTGGATTT	CTCCTGACCCTTCATCCGTA
*Survivin*	TGCAAAGGAGACCAACAACA	AAGCTGGGACAAGTGGCTTA
*NFκB*	GCTTACGGTGGGATTGCATT	GCACAATCTCTAGGCTCGTTTTTAA
*TNFα*	GGCTGCCCCGACTACGT	AGGGCAAGGGCTCTTGATG
*DR5*	AAATGCTGCTGAAGTGGCT	ACTAATAAAGATCCTCTCGGCTC
*BiP*	GACCACCTATTCCTGCGTCGGT	CGCCAATCAGACGCTCCCCT
*Caspase12*	GGAAGGTAGGCAAGAGT	GTAGAAGTAGCGTGTCATA
*GADD34*	TGAATGTTGAGAGAAGAACC	TTGTTTAGAAGTCGCTCTG
*PERK*	GCTTGCTCCCACATCGGATA	TGCGGCAATTCGTCCATCTA
*IRE1*	TTGACTATGCAGCCTCACTTC	AGTTACCACCAGTCCATCGC
*eIF2 α*	ATAGGCGTTTGACCCCACAA	ATCACATACCTGGGTGGAGC
*ATF4*	CCTTCGACCAGTCGGGTTTG	CTGTCCCGGAAAAGGCATCC
*ATF6*	AAGTGAAGAACCATTACTTTATATC	TTTCTGCTGGCTATTTGT
*p53*	TATGACTTTAGGGCTTGTTA	AGCAACTACCAACCCATTC
